# Case Report: Hepatocellular adenoma due to long-term oral stanozolol administration

**DOI:** 10.3389/fmed.2025.1654316

**Published:** 2025-08-20

**Authors:** Ya-Nan Qin, Chang-Ming Tao, Tian-Tian Guo, Jing-Jing Liu, Wei-Chang Luan, Chun-Hua Liu

**Affiliations:** ^1^Department of Infectious Diseases, Liaocheng People's Hospital, Liaocheng, China; ^2^Imaging Department, Liaocheng People's Hospital, Liaocheng, China; ^3^Emergency Department, Liaocheng People's Hospital, Liaocheng, China

**Keywords:** aplastic anemia, stanozolol, androgens, case report, hepatocellular adenoma

## Abstract

This article presents a case of a 15-year-old male with a 6-year history of aplastic anemia treated with long-term oral stanozolol to promote hematopoiesis. Throughout this period, he underwent regular outpatient follow-up assessments of blood and liver function parameters. While abnormal liver function was recorded on several occasions and treated with oral hepatoprotective drugs, no abdominal imaging test was conducted. On this occasion, the patient was admitted to hospital with abdominal pain. Abdominal imaging revealed a liver tumor of undetermined nature. A diagnosis of *β*-catenin-activated hepatocellular adenoma was subsequently confirmed via hepatic perforation biopsy. Considering the high bleeding risk, transcatheter hepatic artery embolization was performed as a preventative measure. Stanozolol was discontinued immediately after diagnosis and replaced with platelet-boosting therapy using romiplostim. A repeat abdominal CT scan performed 4 months after discontinuation of the drug showed a significant reduction in lesion size, which continued to be closely monitored. Hepatocellular adenoma is a rare clinical scenario. This case, supported by complete data and gold-standard pathologic diagnosis, provided valuable insights, suggesting that patients on long-term androgen therapy with aplastic anemia constituted a high-risk group for hepatocellular adenoma, and highlighted the need to optimize management strategies.

## Introduction

Stanozolol is commonly used in the treatment of aplastic anemia (AA) due to its ability to stimulate bone marrow hematopoiesis. However, its long-term application has been linked to the development of hepatocellular adenoma (HCA). HCA is extremely rare in children, accounting for only 2% of all pediatric liver tumors (1). Although benign, HCA carries a potential risk of spontaneous rupture, hemorrhage, and malignant transformation. In this article, we report a case of hepatocellular adenoma secondary to long-term oral stanozolol therapy in a patient with aplastic anemia. The clinical features, diagnosis and treatment strategies are discussed, alongside a review of the literature, with a view to providing a guidance for clinical diagnosis and disease management.

## Case presentation

A 15-year-old male patient was admitted to the hospital on December 2, 2024 with a complaint of “paroxysmal right upper abdominal pain for one day.” Initially, the pain was dull and lingered for more than 10 min before resolving on its own, so it was dismissed. However, this morning, it intensified abruptly without an obvious trigger. Flexing forward or lying on the side only provided partial relief, prompting the patient to seek emergency care. Previously diagnosed with aplastic anemia in December 2018, the patient had been receiving long-term immunosuppressive therapy (cyclosporine 50 mg tid, which was adjusted to tacrolimus 0.5 mg tid in June 2023 due to developing renal insufficiency). Concurrently, the patient continued to take stanozolol (2 mg tid) for 6 years, during which time he additionally received adjuvant therapy with avatrombopag, caffeic acid, and compound glycyrrhizic acid tablets. Physical examination findings on admission included: pulse (P) 115 beats/min, blood pressure (BP) 149/91 mmHg, positive percussion pain in the liver area, mild pressure pain on the right side of the abdomen, no rebound pain, and no other significant positive sign.

**Figure 1:**
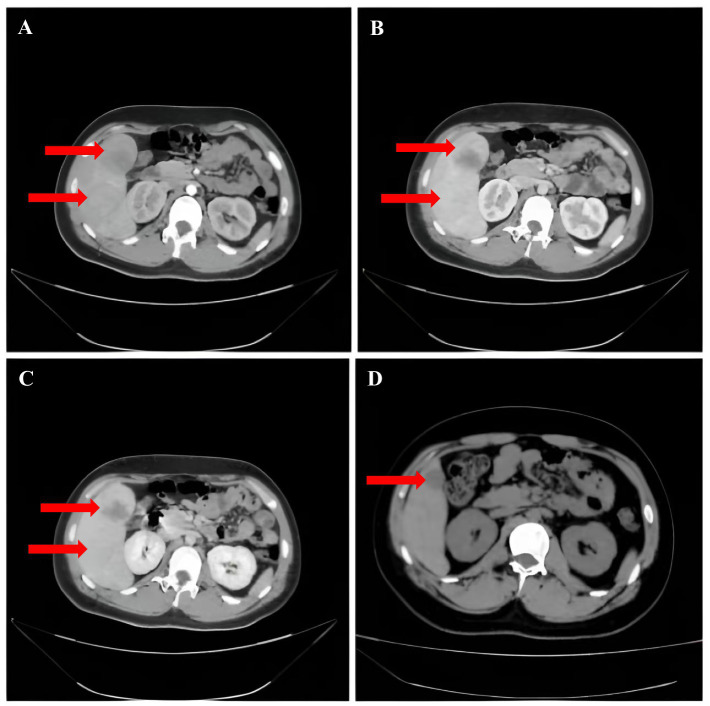
**(A)** Significant enhancement in the arterial phase after enhancement, with a ring-shaped low-density envelope observed at the edge. **(B,C)** Enhancement in the portal and equilibrium phases is slightly higher than that in liver parenchyma. **(D)** Multiple roundish mixed-density nodules in the right liver.

**Figure 2:**
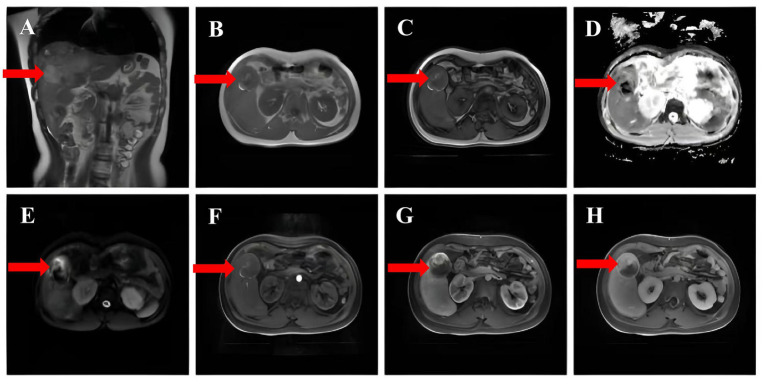
**(A)** Fast echo spin (FSE) showing mixed hypointense signals. **(B,C)** No significant signal reduction in the same inverse phase was observed. **(D,E)** DWI and ADC revealed no significant diffusion restriction. **(F,G,H)** Contrast-enhanced MRI scans with delayed phase enhancement.

Complementary examinations: A routine blood test revealed a platelet (PLT) count of 36.00 × 10^9^/L (reference range: 125–350 × 10^9^/L). Liver function tests showed levels of alanine aminotransferase (ALT) of 43 U/L (7–40 U/L), aspartate aminotransferase (AST) of 32 U/L (13–35 U/L), and Y-glutamyltranspeptidases (y-GT) of 158 U/L (7–45 U/L). Kidney function assessment demonstrated a serum creatinine level of 100.5 μmol/L (41–81 μmol/L). Coagulation studies revealed D-dimer levels of 4.01 mg/L (0–0.5 mg/L). The alpha-fetoprotein (AFP) content was normal and serum levels of protein induced by vitamin K absence or antagonist II (PIVKA-II) were 271.44 U/mL (0–40 ng/mL). Hepatitis virus markers, including hepatitis B and C, were negative. Targeted genetic testing for metabolic disorders was not performed. Contrast-enhanced computed tomography of the upper abdomen disclosed multiple iso-low mixed density foci in the liver. The largest lesion measured about 5.7 cm in diameter, with ill-defined borders. A number of lesions displayed segregation-like changes and inhomogeneous enhancement after enhancement, with CT values of 49–78 H (Figures 1A–C). Hepatobiliary contrast-enhanced MRI showed that the liver was obviously enlarged, with multiple, irregular prolonged T1 and slightly prolonged T2 signals. On diffusion-weighted imaging (DWI), some of the lesions showed restricted dispersion and the arterial phase of the dynamic enhancement scan displayed a ring shape. A dynamic enhancement scan showed ring-shaped and nodular mild enhancement in the arterial phase, progressive enhancement in the portal phase, and signal reduction in the delayed phase. Additionally, curved long T1 and long T2 signal shadows were observed in the perihepatic and peri-splenic areas (Figure 2). On December 11, 2024, due to the presence of liver lesions of unknown etiology, the patient’s guardian expressed a willingness to cooperate fully in order to establish a clear diagnosis, the patient underwent hepatic puncture biopsy (right anterior lobe of the liver) under local anesthesia. Despite the administration of Atriplopa prior to the procedure, the platelet count remained below 50 × 10^9^/L and did not reach the target threshold. Histological analysis revealed a proliferative hepatocellular lesion with moderate differentiation, structural disorder, and localized pseudo-glandular ductal structures. The immunohistochemistry profile was as follows: GPC-3 (−), GS (+), Hsp70 (+), Arg-1 (−), HepPar-1 (hepatocyte +), CD34 (vascular +), CK19 (bile duct +), Ki-67 positivity 5%, *β*-catenin (nuclear +), CD10 (+), and special reticulofibrillary staining (+). These findings were consistent with a diagnosis of *β*-catenin-activated hepatocellular adenoma.

Treatment and regression: Upon confirmation of diagnosis, stanozolol was immediately discontinued and replaced with romiplostim. Due to the high risk of bleeding, transarterial chemoembolization via the hepatic artery was performed alongside the percutaneous liver biopsy.

Follow-up: The patient and guardian exhibited poor adherence, reappearing only 4 months later. Laboratory panels showed a further drop in platelet count relative to pre-treatment values. A CT scan of the upper abdomen revealed a marked reduction in lesion size 4 months after the procedure (Figure 1D). Due to financial constraints, the guardian chose to stop romiplostim and start avatrombopag again. The patient is also taking oral traditional Chinese medicine prescribed by another institution. Close outpatient surveillance is ongoing.

A brief case summary is shown in Table 1.

**Table 1:** Case summary.

Date	Event category	Specific details
December 01, 2018	Past medical history	A diagnosis of aplastic anemia (AA) was made, and the patient was started on cyclosporine (50 mg tid po) and stanozolol (2 mg tid po), as well as long-term TPO-receptor agonists and adjuvant drugs.
June 15, 2023	Past medical history	Due to renal insufficiency, the immunosuppressant medication was switched from cyclosporine to tacrolimus (0.5 mg three times daily); stanozolol was continued.
December 02, 2024	Admission day	Chief complaint: Aroxysmal right upper abdominal pain for 1 day. Vital signs: Pulse: 115 bpm; Blood pressure: 149/91 mmHg; Mild pressure pain on the right side of the abdomen.
December 02, 2024	Laboratory and imaging examinations	Platelets 36 × 10̂9/L, γ-GT↑, creatinine 100.5 μmol/ L, PIVKA-II 271.44 U/mL. Contrast-enhanced CT: Multiple hepatic masses, largest measuring 5.7 cm. MRI: multiple abnormal hepatic signals with focal hemorrhage suggestive of a neoplastic lesion. Biopsy recommended.
December 03, 2024	Consultation	Hematology: No treatment adjustment. Nephrology: Continue the tacrolimus treatment with Xanthone capsules.
December 11, 2024	Diagnosis and therapy	Ultrasound-guided percutaneous liver biopsy of the right anterior lobe was performed under local anesthesia. Due to the high risk of bleeding, hepatic arterial chemoembolization (TACE) was performed immediately after the biopsy.
December 16, 2024	Diagnosis	Histopathology and immunohistochemistry confirmed a *β*-catenin-activated hepatocellular adenoma (*β*-catenin nuclear-positive and Ki-67 at 5%).
December 16, 2024	Intervention	Stanozolol was immediately discontinued and replaced with romiplostim.
April 13, 2025	Follow-up	A four-month post-procedure contrast-enhanced CT scan revealed a significant reduction in the size of the lesion.

## Discussion

Hepatocellular adenomas are rare, benign liver tumors with significant differences in incidence based on sex and age. In adults, the incidence is about 3–4 per 100,000, occurring predominantly in women of childbearing age, and typically arising from otherwise healthy livers. In contrast, the incidence in children is extremely low, estimated at only 1 per 1,000,000, and is most commonly associated with inherited metabolic disorders or an abnormal hepatic background (2, 3). Recognized risk factors for development of HCA include prolonged use of oral contraceptives in women, anabolic steroid use, obesity, alcoholism, and diabetes mellitus. In children, inherited disorders, such as glycogen-accumulating diseases, galactosemia, immunodeficiency disorders, congenital hepatic fibrosis, type 3-young adult diabetes mellitus, Fanconi anemia, and other rare metabolic conditions are associated with HCA. In the present case, the patient had a 6-year history of exposure to stanozolol, an anabolic steroid with a primary structure and physiologic effects similar to androgens, which promotes aberrant proliferation of hepatocytes through activation of the *β*-catenin signaling pathway. This mechanism is highly consistent with pathologically proven *β*-catenin-activated HCA (B-HCA), a subtype with a high (46%) malignancy rate that is particularly prevalent in male patients.

Hepatocellular adenomas are usually asymptomatic and are often discovered incidentally during abdominal imaging. Spontaneous tumor rupture or hemorrhage occurs in approximately 15–20% of cases, presenting with acute abdominal pain. (4). In the current case, the patient presented with acute abdominal pain, and imaging evaluation revealed evidence of hemorrhage, highlighting the need for heightened clinical vigilance for asymptomatic patients at potential risk. On CT scans, hepatic adenomas typically exhibit inhomogeneous density, particularly in lesions exceeding 4 cm in diameter. Small lesions in the arterial phase of enhanced CT tend to exhibit homogeneous enhancement while large lesions often show non-homogeneous enhancement due to necrosis. In the present case, enhanced CT showed non-uniform enhancement in the arterial phase, continuous enhancement in the portal phase, delayed enhancement slightly lower than that of surrounding normal hepatic parenchyma, and necrosis and foci of hemorrhage in the lesion, in line with earlier literature reports. MRI (5–7) has been shown to aid in the identification of more than 80% of HCA subtypes, especially Hepatocyte nuclear factor-lɑ HCA (H-HCA) and Inflammatory-type HCA (I-HCA), with a reported specificity of up to 90%. However, differentiation between *β*-HCA and unclassified HCA and hepatocellular carcinoma (HCC) remains a considerable diagnostic challenge. Hepatic puncture biopsy should be performed in cases where hepatic adenomas are atypical on imaging or malignancy is considered based on changes in imaging features. However, androgen-exposed pediatric HCA, in particular, large tumors with a richer intra-tumoral blood supply, have a significant risk of bleeding, which is further amplified in the reentrant population. Wang et al. (8) reported a case of a patient with a 4-year history of oral stanozolol therapy for hepatocellular adenoma, who developed hemorrhagic shock on two occasions during the consultation and treatment due to underestimation of the bleeding risk of the tumor. These events highlight the potentially life-threatening nature of hemorrhagic complications. Similarly, our patient also belonged to the reentrant population and had large, multiple tumors characterized by a rich blood supply and intralesional hemorrhagic foci, posing a high hemorrhagic risk.

Clinicians take the preliminary step of image segmentation (e.g., accurate delineation of the liver and tumor) to optimize diagnosis, staging, and treatment planning and intervention (e.g., surgical resection, radiotherapy, embolization, etc.). Dakua et al. (9) proposed a fully automated left-ventricular contour extraction pipeline that synergistically couples a cantilever-beam physical model with a graph-theoretic random-walk algorithm. This method is fast, accurate, and fully automated. Moreover, we can apply the method to all types of gray-scale images in a clinical setting. Therefore, it can be used for liver tumors, including HCA. Specific implementation is as follows. First, pixel intensities are treated as loads on a virtual cantilever beam, and a 360° sweep is performed to identify pronounced deflection points at the hepatocellular adenoma (HCA)-liver interface. This generates a sparse set of automatic seeds. Second, these seeds are fed as Dirichlet boundaries into random-walk algorithm. This algorithm computes the most probable label for each pixel on a weighted graph. This allows for the accurate delineation of the HCA from the surrounding normal hepatic parenchyma.

For this patient, Imaging reveals hemorrhage and necrosis with a thin pseudocapsule. Heterogeneity increases with size, particularly in *β*-catenin-mutated lesions. Arterial hyperenhancement persists into the portal phase and then gradually washes out, lacking the classic HCC wash-in/wash-out pattern. Precise segmentation is essential for reliably extracting texture, edge sharpness, heterogeneity, and vascular distribution. Background parenchyma easily contaminates texture; Dakua et al.’s (9) proposed noise-robust random-walk algorithm with automatic seed selection preserves statistical homogeneity within the lesion. Edge sharpness is often degraded by low contrast and partial-volume effects. Their adaptive-threshold technique, combined with a cantilever-beam gradient response, enables subpixel edge localization. This yields a more accurate quantification of tumor boundary acuity. Heterogeneity assessment requires subregional partitioning prior to feature extraction. A multi-seed strategy can divide the tumor into viable and necrotic compartments for independent analysis. Vascular metrics use background noise estimation to enhance vessel-to-lesion contrast. This allows for the extraction of key radiomic features, such as vascular invasion grade and vessel-to-tumor distance ratio.

Identifying adenoma subtypes noninvasively remains challenging. Accurate segmentation is the first and arguably the most critical step in any radiogenomic investigation of steroid-induced hepatocellular adenomas (HCAs). Segmentation localizes and stratifies lesions into molecularly coherent compartments, enabling robust genotype–phenotype mapping in HCAs. In the study by Guillaume et al. (10) a multivariable model that integrated MRI-based radiomics, age, and sex was used to noninvasively subtype 83 histologically confirmed hepatocellular adenomas (HCAs). The ASVT radiomics model achieved superior balanced accuracies compared with conventional visual assessment for small-hybrid HCA (sh-HCA) (73.8%), beta-HCA (71.9%), and binary discrimination of intermediate-HCA (I-HCA) versus beta-I-HCA (73%). The study also describes imaging characteristics that include smooth tumor margins and thin, consistent pseudocapsules. *β*-catenin-mutated HCAs tend to be larger and appear more heterogeneous, frequently containing hemorrhage and necrosis. Given these findings, the radiologic phenotype observed in our patient aligns with steroid-induced β-HCA, which is consistent with the published literature. Therefore, combining basic clinical variables with radiomic features improves the noninvasive subtyping of HCAs. This is especially useful for small lesions (less than 5 cm) and patients at high risk of bleeding who would otherwise require a biopsy.

The categorization of segmentation methods has been often subjective; they are generally classified based on their methodology or extent of human intervention. Segmentation methods can also be classified as semi-automatic or automatic based on the extent of human intervention (11). The diagnosis and treatment outcomes could potentially be impacted by segmentation methods. However, the diagnosis of HCA is a complex procedure that may be impacted by tumor morphology, hemorrhage or necrosis, vascular artifacts, malignant or benign nature. Bilic et al. (12)evaluate more than 24 cutting-edge liver and tumor segmentation techniques and determine that a single segmentation algorithm might not always be the optimal choice for segmenting the liver and its tumors. Many studies have validated the accuracy of volumetry of the liver using cross-sectional imaging (13). This method is regarded as the gold standard for liver volume assessment. A high level of agreement was found between CT-based volume index and true liver volume by D. Seppelt et al. (14). The liver volume can be estimated reliably, quickly, and simply using a volume index based on CT diameters. This approach can be recommended for clinical practice. Although manual volumetry is time-consuming and requires an experienced radiologist, this case is a alone case and involves minimal workload. Despite the multifocal lesions, using the largest tumor diameter to estimate tumor volume is a reasonable clinical approach for disease assessment. Serial measurements showed a marked reduction in the largest tumor volume post-treatment, indicating a favorable therapeutic response.

Because hepatocellular adenomas are mostly benign lesions, the majority can be stabilized or undergo regression following the elimination of hormonal stimuli or metabolic risk factors (15, 16). In hormone-driven hepatocellular adenomas, tumor size is significantly correlated with hormone levels, with 50% of tumors achieving a reduction in size or cessation of growth after discontinuation of oral hormone medications (17). The 2024 American College of Gastroenterology guidelines suggest a more aggressive treatment approach in male patients, given the substantially elevated risk of malignancy. Surgical resection is specifically recommended as the first-line treatment, regardless of tumor subtype or size, and preoperative hepatic puncture biopsy is not required (3). This recommendation is based on the high (46%) malignancy rate associated with *β*-catenin-mutated HCA and the observation that male patients often present with combined risk factors, such as metabolic abnormalities. In this case, the patient was an adolescent male experiencing abdominal pain and discomfort. Imaging revealed multiple liver lesions; the largest measured 5.7 cm in diameter and exhibited localized hemorrhagic signals. Pathological analysis confirmed the lesions to be *β*-catenin-activated. There were multiple indications for surgical intervention; however, the patient had a low platelet count, and platelet-boosting therapy was deemed ineffective.

Transcatheter hepatic artery embolization (TAE) or radiofrequency ablation (RFA) serve as viable alternative treatment options for non-operative patients. A systematic review of 851 patients with hepatocellular adenoma (HA) revealed that 151 patients (17.7%) underwent transarterial embolization (TAE). Among those patients, tumor shrinkage was achieved in 75% and complete disappearance in 10%. (18). Kazuhide Takata presented a case study of a patient with Cornelia de Lange syndrome (CdLS) and a congenital portosystemic shunt (CPSS), who also presented with hepatocellular adenomas (HCAs). Following transcatheter arterial embolization (TAE), the lesions regressed significantly, demonstrating the efficacy of TAE in treating CPSS-induced HCAs (19). TAE is particularly effective for cases with rupture or active bleeding, with a high success rate for hemostasis (20), offering both diagnostic and therapeutic benefits. However, hepatic puncture biopsy is required to clarify the presence or absence of malignancy prior to initiation of treatment. Studies have shown that TACE after an emergency hepatic puncture reduces the risk of post-puncture bleeding or hemorrhagic shock. The patient presented with abdominal pain caused by spontaneous rupture and hemorrhaging. TACE was performed to stop the bleeding and alleviate the discomfort. It is also employed for tumor therapy.

Patients require outpatient follow-up one to 2 months after surgery to assess the extent of tumor necrosis and residual active foci. This assessment informs decisions about secondary TACE or surgical resection, depending on the residual active volume after surgery and the emergence of new foci. The patient was discharged from the hospital with decreased platelets compared to the time of admission. This finding suggests that the criteria for hematologic remission (HR) had not been met, as defined by platelets ≥50 × 10^9^/L and transfusion independence. Thus, the patient was judged to be a “non-responder” and should be considered for upgraded immunosuppressive therapy or allogeneic hematopoietic stem cell transplantation (allo-HSCT) (21). In the context of traditional Chinese medicine, there is a theoretical possibility of reactivating the *β*-catenin pathway, which could result in a rebound or malignant transformation of the lesion.

Several risk factors for HCA malignancy have been identified in previous studies, including advanced age, male sex, anabolic steroid use, metabolic syndrome, tumor diameter >5 cm and *β*-catenin activation subtype (22). Our patient exhibited multiple risk features despite his young age: adolescent male, 6-year history of stanozolol exposure, and pathologically confirmed diagnosis of *β*-catenin mutant HCA (immunohistochemistry disclosed diffuse GS positivity and *β*-catenin nuclear positivity). Additionally, the patient showed elevated PIVKA-II (271.44 U/mL) and a Ki-67 proliferative index of 5%, indicative of active proliferative potential and potential malignant tendency. Notably, the malignant transformation of HCA is often characterized by insidious progression. Gordon et al. (23) reported a case showing progression of HCA to hepatocellular carcinoma (HCC) after a latency period of up to 20 years, with the malignancy occurring in the same anatomical location as the original HCA lesion. In another study (24), an elderly male patient on long-term danazol therapy (19 years) progressed from HCA to HCC in only 8 months. These case reports highlight the critical need for rigorous and sustained long-term surveillance in high-risk patients. Specific recommended strategies for follow-up include: imaging (contrast-enhanced MR examination every 3 months is recommended and early elution of arterial enhancement is a significant feature of malignant transformation (25, 26), which can be extended to every 6 months for those with no progression after 2 years); tumor markers (combined testing of AFP and PIVKA-Il is recommended, of which PIVKA-II has a higher predictive value in detecting malignant transformation of *β*-catenin mutant HCA); pathological reassessment (repeat biopsy should be considered for rapidly growing lesions in follow-up to exclude focal carcinoma). A notable feature of this case is the co-existence of aplastic anemia, which necessitates heightened vigilance for the malignant transformation of HCA, while taking into account the safety of myelosuppressive therapy. A multidisciplinary collaborative approach incorporating hematology, hepatology, and imaging is essential to develop a personalized follow-up intervention plan that optimizes both treatment efficacy and patient safety.

### Limitations

Finally, the therapeutic choices were generally reasonable and effective under the dual diagnosis of *β*-catenin-activated hepatocellular adenoma (HCA) and aplastic anemia (AA). However, there are still some shortcomings and room for improvement. First, the adolescent male patient had been receiving oral stanozolol for aplastic anemia over a 6-year consecutive period, during which regular monitoring of liver function revealed abnormalities on several occasions (specifically, persistent fluctuating elevation of ALT/AST since 2020). However, the supervising pediatrician only attributed this finding to simple pharmacological hepatic damage and prescribed liver-protecting medication to treat the symptomatic condition without pursuing further abdominal imaging, which led to a delayed diagnosis of hepatocellular adenoma. Furthermore, the hematology consultation after admission failed to propose timely adjustment of the treatment plan. This omission reflects cognitive limited cognitive awareness of the tumorigenic mechanisms of long-term androgenic drugs in clinical practice. Secondly, a systematic follow-up protocol was not implemented after TACE. This resulted in a higher potential for overlooking early residual or *de novo* lesions. Financial constraints further interrupted therapy, leading to platelet fluctuations, uncertain efficacy, and an increased risk of bleeding. Rather than merely reacting after drug withdrawal, physicians should proactively assess the economic status of their patients’ families. Furthermore, the composition of the prescribed traditional Chinese medicine is not well understood, and hepatotoxicity is possible. Third, there is an absence of documentation regarding multidisciplinary team involvement.

## Conclusion

This case revealed a clear association between long-term use of stanozolol and the development of hepatocellular adenoma, suggesting that patients with AA receiving long-term androgenic drugs are at increased risk of developing hepatic sex hormone-related tumors. Routine surveillance is strongly recommended for such patients, including tests on liver function, AFP (combined with the PIVKA-II assay, if necessary), and abdominal ultrasound. In cases where intrahepatic nodules or masses are detected, androgen therapy should be discontinued and closely reviewed. Where necessary, invasive procedures can be performed to further determine the nature of the lesions. For patients presenting with hepatocellular adenoma with thrombocytopenia, proper caution should be taken due to the elevated risk of spontaneous tumor rupture and bleeding, and platelet boosting or combination with interventional embolization before puncture biopsy considered to minimize the likelihood of hemorrhage.

## Data Availability

The original contributions presented in the study are included in the article/supplementary material, further inquiries can be directed to the corresponding authors.
